# Association between nighttime-daytime sleep patterns and chronic diseases in Chinese elderly population: a community-based cross-sectional study

**DOI:** 10.1186/s12877-019-1136-9

**Published:** 2019-04-29

**Authors:** Shuo Zhang, Li Xie, Herbert Yu, Weituo Zhang, Biyun Qian

**Affiliations:** 1 Hongqiao International Institute of Medicine, Shanghai Tongren Hospital & Faculty of Public Health, Shanghai Jiao Tong University School of Medicine, No. 227, South Chongqing Road, Shanghai, 200025 China; 2 Clinical research center, Shanghai Jiao Tong University School of Medicine, No. 227, South Chongqing Road, Shanghai, 200025 China; 3 Cancer Epidemiology Program, University of Hawaii Cancer Center, 701 Ilalo Street, Honolulu, HI 96813 USA

**Keywords:** Sleep pattern, Napping, Chronic diseases, Elderly

## Abstract

**Background:**

This study aimed to assess the relationship between specific nighttime-daytime sleep patterns and prevalence of different chronic diseases in an elderly population.

**Methods:**

We conducted a community-based cross-sectional study in 4150 elderly Chinese, with an average age of 74 years. Sleep-related variables (nighttime sleep duration, daytime napping and duration) and chronic disease status, including diabetes, cardiovascular diseases (CVD), dyslipidemia cancer and arthritis were collected for the study. Multivariable logistic regression models were used to analyze the relationship between nighttime-daytime sleep patterns and prevalence of chronic diseases.

**Results:**

Overall prevalence of any of chronic diseases was 83.8%. Nighttime-daytime sleep patterns were defined according to nighttime sleep duration and habitual nappers/non-nappers. Taking the nighttime-daytime sleep pattern “short nighttime sleep with daytime napping” as reference, those with “long nighttime sleep without daytime napping” had higher prevalence of diabetes [OR and 95% CI, 1.35 (1.01–1.80)] and lower prevalence of arthritis [OR and 95% CI, 0.46 (0.33–0.63)]. And those with “long nighttime sleep with daytime napping” had higher prevalence of diabetes [OR and 95% CI, 1.36 (1.05–1.78)] while lower prevalence of cancer [OR and 95% CI, 0.48 (0.26–0.85)] and arthritis [OR and 95% CI, 0.67 (0.51–0.86)]. Further, in habitual nappers, subjects were classified according to duration of nighttime sleep and daytime naps. Compared to “short nighttime sleep with long daytime napping”, individuals with “long nighttime sleep with short daytime napping” had significantly positive association with diabetes prevalence [OR and 95% CI, 1.73 (1.15–2.68)] while border-significantly and significantly negative association with cancer [OR and 95% CI, 0.49 (0.23–1.07)] and arthritis [OR and 95% CI, 0.64 (0.44–0.94)], respectively.

**Conclusions:**

Elderly individuals with chronic diseases had different nighttime-daytime sleep patterns, and understanding these relationships may help to guide the management of chronic diseases.

**Electronic supplementary material:**

The online version of this article (10.1186/s12877-019-1136-9) contains supplementary material, which is available to authorized users.

## Background

Chronic diseases constitute a major challenge to global public health with respect to quality of life and longevity [1]. In China, more than 80% of deaths and 70% of disability-adjusted life-years lost were reported due to chronic diseases in 2008 [2, 3]. Chinese populations are rapidly aging in recent decades, which intensifies the impact of chronic diseases [4, 5]. The number of people diagnosed with chronic diseases increases with age and over the years. It was reported that the proportion of those seniors aged 65 years or older suffering from one or more chronic diseases rose from 86.9 to 92.2% in the US from 1998 to 2009 [6]. In addition, several chronic diseases often occur concurrently in elderly people, which makes the management of these disease more challenging [7]. A large population-based study conducted in China showed significant correlations between chronic diseases, such as hypertension and dyslipidemia, dyslipidemia and diabetes, diabetes and arthritis, which makes the intervention and health promotion more difficult to carry out among the elderly populations [8].

Sleep physiology undergoes significant changes across the lifespan, and the distributions of sleep duration vary with age [9, 10]. Numerous health problems prevalent in the elderly may also influence sleep duration [9]. Thus, the relationship between sleep duration and chronic diseases differs for the elderly and middle-aged adults. Sleep habits may be an important indicator of chronic diseases including diabetes, hypertension, dyslipidemia, coronary heart diseases and cancer, though research findings have been mixed [11–14]. Previous studies often emphasized on the all-day-long sleep duration with consideration of the distribution of nighttime and daytime [15]. Daytime napping is regarded as one component of a healthy lifestyle, especially in Latin America and Mediterranean countries [16, 17]. In China, daytime napping is a popular sleep behavior. Fang et al. reported that the prevalence of daytime napping was about 68.6% in a middle-aged Chinese population [16]. Further, significant relationship was found between nighttime sleep and daytime napping [18]. Therefore, more research is needed to understand what patterns of nighttime and daytime sleep are healthy, and for whom.

Research design is very critical in exploring the relationship between sleep patterns and chronic diseases [19–21]. Altered sleep may affect or result from a chronic disease. Elucidating the association between specific sleep patterns, and different chronic diseases, has significant implication in public health and management of chronic diseases. Here, we reported a large community-based cross-sectional study which investigated the relationship between nighttime and daytime sleep patterns and chronic diseases in a Chinese elderly population.

## Methods

### Study population

**Fig. 1:**
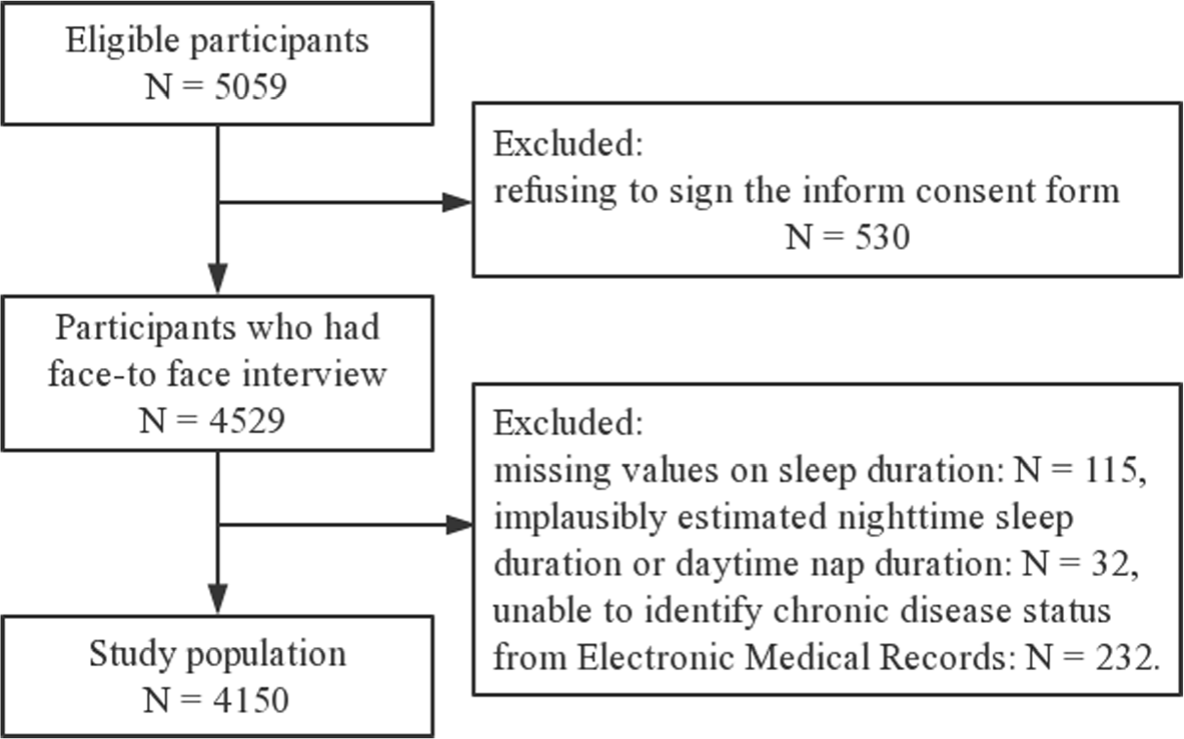
Flow chart of the study population enrollment

A community-based cross-sectional study was conducted involving 4150 adults living in urban Shanghai, China. Participants were enrolled in a prospective cohort (ChiCTR-EOC-16010110), and this study came from the baseline data. We included participants who were permanent residents, aged over 65 years, living in the community of urban Shanghai. All study participants signed a written informed consent form before enrollment. The study was conducted in accordance with the Declaration of Helsinki and was approved by the Ethical Review Committee of the School of Public Health at Shanghai Jiao Tong University. Flow chart of study population recruitment is shown in Fig. 1.

### Data collection and measurement

All participants underwent a face-to-face interview conducted by trained interviewers. Information on demographic and anthropometric factors (e.g., age, gender, BMI), medical history (e.g., diabetes, dyslipidemia, cardiovascular diseases, cancer, and other chronic diseases), and lifestyle characteristics (e.g., physical activity, smoking and drinking status) was collected using a validated structure questionnaire.

### Sleep-related variables

Self-reported sleep habits were obtained through the following questions: “How long, on average, do you sleep at night?”, “Do you have a habit of daytime napping?” and “How long, on average, do you spend on daytime napping?” Total sleep duration was calculated by adding nighttime sleep hours with daytime napping hours (if no daytime napping, daytime napping duration equals 0). For data analysis, we first define the nighttime-daytime sleep patterns according to nighttime sleep duration plus habitual nappers versus non-nappers for the whole population. Then, we defined the nighttime-daytime sleep duration patterns according to nighttime sleep duration and daytime napping duration in the habitual nappers.

### Chronic diseases

In this study, chronic diseases refer to diabetes, CVD (including coronary heart diseases, hypertension and stroke), dyslipidemia (including hyperlipidemia and non-alcoholic fatty liver [22]), cancer (any type) and arthritis. All study subjects participated were asked to complete a medical history questionnaire and underwent a physical exam and routine blood and urine laboratory tests. All chronic diseases were based on the diagnosis of physicians.

### Other covariates of interest

Other covariates included in the study were age, gender, body mass index (BMI), physical activity level (PAL), family income, and number of family members. Moreover, our study also collected information on many health-related behaviors. Smoking status and alcohol consumption were collected and used to adjust for confounding in association analysis [23]. Smoking status was classified as current smoker, ex-smoker, and never smoked. Current smokers were defined as having smoked at least one cigarette per day for more than one year [24]. Ex-smokers were defined as those who had completely quit smoking, but had smoked > 100 cigarettes in their lifetime. Alcohol assumption was classified as never drinking or drinking less than once a month, past alcohol drinker (> 1/month), and current alcohol drinker (> 1/month). PAL was measured using the International Physical Activity Questionnaire (IPAQ). Height and weight were measured by the nurses in the community hospitals at enrollment. BMI was calculated as weight (kg)/height (m^2^).

### Statistical analysis

Descriptive statistics were calculated first, including means, standard deviations (SD), and percentages. Differences in demographic characteristics and potential risk factors of chronic disease between sleep duration groups and sleep pattern groups were analyzed using the chi-square test for categorized variables and the Student’s t-test for continuous variables. Unconditional logistic regression models were used to assess the strength of associations between sleep patterns and prevalence of chronic diseases. To investigate if the association between nighttime-daytime sleep patterns and prevalence of chronic diseases are independent from confounding variables, two models were fitted: The crude model and the multivariable adjusted model, which was adjusted for age, gender, BMI, PAL, smoking status, alcohol consumption and arthritis (not adjusted for arthritis in the association between sleep patterns and arthritis). All statistical procedures were performed using R software 3.4.4. Statistical tests were two-sided with *P* < 0.05 set as significance level.

## Results

### Characteristics of the population by nighttime sleep, daytime napping and total sleep

Table 1 shows the demographic characteristics of study participants according to nighttime sleep, daytime napping and all-day-long total sleep. A total of 4150 adults living in urban Shanghai completed the survey. Overall, the mean age of the participants was 74 years, and 55.6% of the participants were female. With regard to specific types of chronic diseases, CVD has the highest prevalence (64.3%), followed by dyslipidemia (31.3%), diabetes (20.0%), arthritis (14.6%) and cancer (4.3%). Among the entire study population, mean nighttime sleep duration and all-day-long total sleep duration were 6.64 h and 7.10 h, respectively, and mean daytime napping duration was 48.22 min with 56.6% of the study subjects having habitual daytime napping.

**Table 1:** Characteristics of the population by nighttime sleep, daytime napping and total sleep

	Nighttime sleep		*P*-value	Daytime napping			*P*-value	Total sleep		*P*-value
	≤ 6.5 h	> 6.5 h		Non-nappers	≤ 45 min	> 45 min		≤ 7.0 h	> 7.0 h	
No. subjects (%)	2252 (47.5)	2488 (52.5)		2112 (43.4)	1752 (36.0)	1004 (20.6)		2642 (55.7)	2098 (44.3)	
Age (years)	74.30 (6.90)	73.65 (7.18)	0.002	73.41 (7.03)	74.42 (7.10)	74.24 (6.98)	< 0.001	74.07 (6.88)	73.81 (7.27)	0.215
Female (%)	1306 (60.2)	1238 (51.4)	< 0.001	1169 (57.8)	924 (55.0)	516 (52.3)	0.014	1519 (59.7)	1025 (50.5)	< 0.001
BMI (kg/m^2^)	24.15 (4.29)	26.30 (61.86)	0.119	27.79 (86.89)	24.26 (4.46)	24.35 (4.64)	0.135	24.24 (4.75)	26.61 (67.59)	0.087
Physical activity (Met·h/week)	38.99 (131.08)	37.72 (159.27)	0.801	41.52 (198.05)	38.90 (110.72)	29.74 (36.14)	0.215	39.30 (148.82)	37.12 (143.81)	0.666
Number of family members	2.58 (1.35)	2.55 (1.19)	0.391	2.60 (1.32)	2.54 (1.22)	2.57 (1.26)	0.315	2.59 (1.33)	2.55 (1.18)	0.277
Family income (yuan, RMB)	6965.84 (12,668.88)	7253.11 (9542.72)	0.424	6889.40 (7815.00)	7011.88 (7484.94)	7733.63 (19,094.86)	0.188	6891.92 (11,754.57)	7393.42 (10,298.42)	0.164
Smoking status (%)			< 0.001				0.375			< 0.001
Non-smoker	1808 (85.4)	1868 (80.1)		1578 (81.8)	1405 (84.0)	750 (82.1)		2109 (85.0)	1567 (79.6)	
Ex-smoker	172 (8.1)	263 (11.3)		205 (10.6)	147 (8.8)	87 (9.5)		218 (8.8)	217 (11.0)	
Current smoker	135 (6.4)	201 (8.6)		146 (7.6)	120 (7.2)	76 (8.3)		152 (6.1)	184 (9.3)	
Alcohol consumption (%)			0.525				0.444			0.388
None or < once/month	1960 (89.1)	2156 (88.2)		1778 (88.2)	1508 (88.0)	887 (90.1)		2304 (89.2)	1812 (87.9)	
Past alcohol drinker (>once/month)	201 (9.1)	247 (10.1)		204 (10.1)	168 (9.8)	84 (8.5)		239 (9.2)	209 (10.1)	
Current alcohol drinker (>once/month)	39 (1.8)	41 (1.7)		33 (1.6)	36 (2.1)	14 (1.4)		41 (1.6)	39 (1.9)	
Any of chronic diseases (%)	1685 (85.9)	1772 (82.0)	0.001	1501 (83.0)	1279 (82.3)	740 (88.2)	< 0.001	1938 (84.9)	1519 (82.6)	0.051
Diabetes (%)	363 (17.8)	451 (19.9)	0.085	366 (19.5)	298 (18.9)	164 (17.9)	0.597	435 (18.2)	379 (19.8)	0.194
CVD (%)	1277 (56.7)	1348 (54.2)	0.086	1106 (52.4)	982 (56.1)	582 (58.0)	0.006	1447 (54.8)	1178 (56.1)	0.358
Dyslipidemia (%)	634 (28.2)	638 (25.6)	0.056	542 (25.7)	484 (27.6)	273 (27.2)	0.359	724 (27.4)	548 (26.1)	0.338
Cancer (%)	93 (4.7)	82 (3.8)	0.145	78 (4.3)	57 (3.7)	44 (5.0)	0.329	102 (4.4)	73 (4.0)	0.518
Arthritis (%)	389 (17.3)	307 (12.3)	< 0.001	266 (12.6)	283 (16.2)	160 (15.9)	0.003	424 (16.0)	272 (13.0)	0.003

Unless indicated otherwise, data are given as the mean ± SD or as number and percentages

*Abbreviations: BMI* body mass index, *PAL* physical activity level

Nighttime sleep, daytime napping and total sleep were all significantly associated with gender. Females were more likely to have shorter nighttime sleep, shorter or no daytime napping and shorter total sleep hours. Nighttime sleep and daytime napping were significantly associated with age. Individuals with short nighttime sleep were older than those with long nighttime sleep, and participants who had daytime napping were older than non-nappers.

Prevalence of any of the chronic disease was found to be significantly higher in participants with short nighttime sleep and long daytime napping, while was not found to be different by total sleep duration group.

### Characteristics of the population by nighttime-daytime sleep patterns

As no significant difference was found between total sleep groups, we further evaluated the nighttime-daytime sleep patterns according to nighttime sleep duration with or without habitual nappers or non-nappers. Nighttime-daytime sleep patterns included: Short nighttime sleep with daytime napping [*N* = 1314 (27.7%)]; Short nighttime sleep without daytime napping [*N* = 938 (19.8%)]; Long nighttime sleep with daytime napping [*N* = 1427 (30.1%)]; Long nighttime sleep without daytime napping [*N* = 1061 (22.4%)]. Nighttime-daytime sleep patterns were significantly associated with age, gender, number of family members and smoking status. Individuals with the pattern of “short nighttime sleep with daytime napping” were slightly older. Females were more likely to have the pattern of “short nighttime sleep without daytime napping”. There was significant difference between nighttime-daytime sleep patterns and the prevalence of any of chronic diseases, prevalence of CVD, dyslipidemia and arthritis. Participants with “long nighttime sleep without daytime napping” had the lowest prevalence of any of chronic diseases, CVD, dyslipidemia and arthritis. No differences were found in sleep pattern for diabetes and cancer (Table 2).

**Table 2:** Characteristics of the population by nighttime-daytime sleep patterns

Variables	Short nighttime sleep with daytime napping	Short nighttime sleep without daytime napping	Long nighttime sleep with daytime napping	Long nighttime sleep without daytime napping	*P*-value
No. subjects (%)	1314	938	1427	1061	
Age (years)	74.56 (6.89)	73.92 (6.91)	74.17 (7.21)	72.95 (7.09)	< 0.001
Female (%)	715 (56.2)	591 (66.0)	717 (52.0)	521 (50.7)	< 0.001
BMI (kg/m^2^)	24.17 (4.81)	24.12 (3.41)	24.42 (4.24)	28.78 (94.06)	0.055
Physical activity (Met·h/week)	37.88 (113.01)	40.65 (154.42)	33.65 (67.96)	43.41 (233.33)	0.549
Number of family members	2.61 (1.31)	2.54 (1.40)	2.49 (1.15)	2.64 (1.24)	0.014
Family income (yuan, RMB)	7326.32 (15,985.04)	6459.34 (5168.58)	7230.51 (9437.45)	7284.66 (9693.30)	0.344
Smoking status (%)					< 0.001
Non-smoker	1050 (85.4)	758 (85.5)	1091 (81.3)	777 (78.5)	
Ex-smoker	94 (7.6)	78 (8.8)	139 (10.4)	124 (12.5)	
Current smoker	84 (6.8)	51 (5.7)	112 (8.3)	89 (9.0)	
Alcohol consumption (%)					0.784
None or < once/month	1144 (89.4)	816 (88.6)	1239 (88.2)	917 (88.1)	
Past alcohol drinker (>once/month)	113 (8.8)	88 (9.6)	137 (9.8)	110 (10.6)	
Current alcohol drinker (>once/month)	22 (1.7)	17 (1.8)	27 (1.9)	14 (1.3)	
Any of chronic diseases (%)	965 (85.6)	720 (86.2)	1047 (83.6)	725 (79.8)	0.001
Diabetes (%)	201 (17.0)	162 (18.9)	261 (20.1)	190 (19.8)	0.233
CVD (%)	747 (56.8)	530 (56.5)	813 (57.0)	535 (50.4)	0.003
Dyslipidemia (%)	361 (27.5)	273 (29.1)	393 (27.5)	245 (23.1)	0.014
Cancer (%)	58 (5.1)	35 (4.1)	43 (3.4)	39 (4.2)	0.231
Arthritis (%)	237 (18.0)	152 (16.2)	205 (14.4)	102 (9.6)	< 0.001

Unless indicated otherwise, data are given as the mean ± SD or as number and percentages

*Abbreviations: BMI* body mass index, *PAL* physical activity level

### Association between nighttime-daytime sleep patterns and prevalence of different chronic diseases

**Fig. 2:**
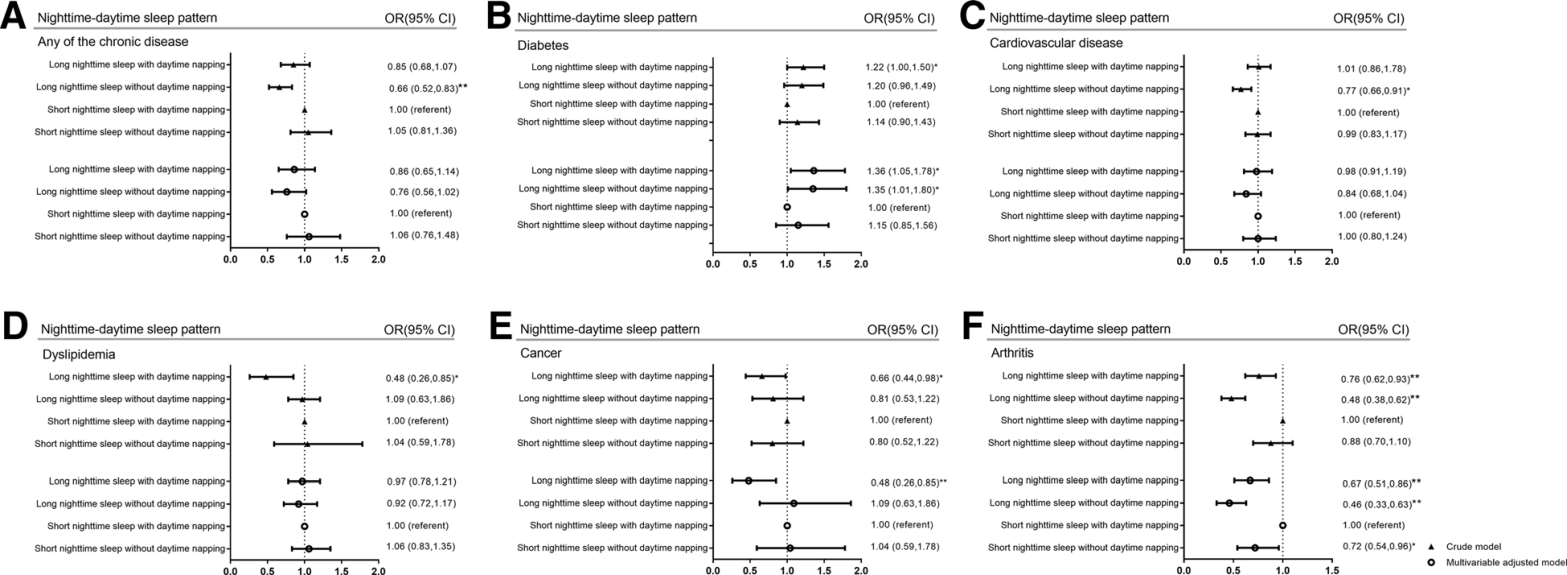
Association between nighttime-daytime sleep patterns and prevalence of individual chronic diseases. Association between nighttime-daytime sleep patterns and prevalence of (**a**) any of the chronic disease, (**b**) diabetes, (**c**) cardiovascular diseases, (**d**) dyslipidemia, (**e**) cancer, (**f**) arthritis in the whole population. * *P* < 0.05, ** *P* < 0.01. Multivariable adjusted factors: adjusted for age, gender, BMI, PAL, smoking status, alcohol consumption and arthritis (not adjusted for arthritis in the association between sleep patterns and arthritis)

As indicated in Fig. 2, multivariable logistic regression models were used to analyze the association between the nighttime-daytime sleep patterns and chronic diseases adjusted for covariates and confounding factors including gender, age, BMI, PAL, smoking status, alcohol consumption and arthritis. As previous studies indicated that decreased sleep duration and increased daytime napping happened more often among the elderly with an average of sleeping duration of 5–7 h/day [10], we used the pattern of “short nighttime sleep with daytime napping” as reference group. In the multivariable adjusted logistic regression model, compared with the reference, people with “long nighttime sleep without daytime napping” had higher prevalence of diabetes (OR 1.35; 95% CI 1.01–1.80, *P* = 0.042) and lower prevalence of arthritis (OR 0.46; 95% CI 0.33–0.63, *P* < 0.001). In addition, people with “long nighttime sleep with daytime napping” had higher prevalence of diabetes (OR 1.36; 95% CI 1.05–1.78, *P* = 0.022), while lower prevalence of cancer (OR 0.48; 95% CI 0.26–0.85, *P* = 0.014) and arthritis (OR 0.67; 95% CI 0.51–0.86, *P* = 0.002). However, no significant association was observed for CVD and dyslipidemia.

**Fig. 3:**
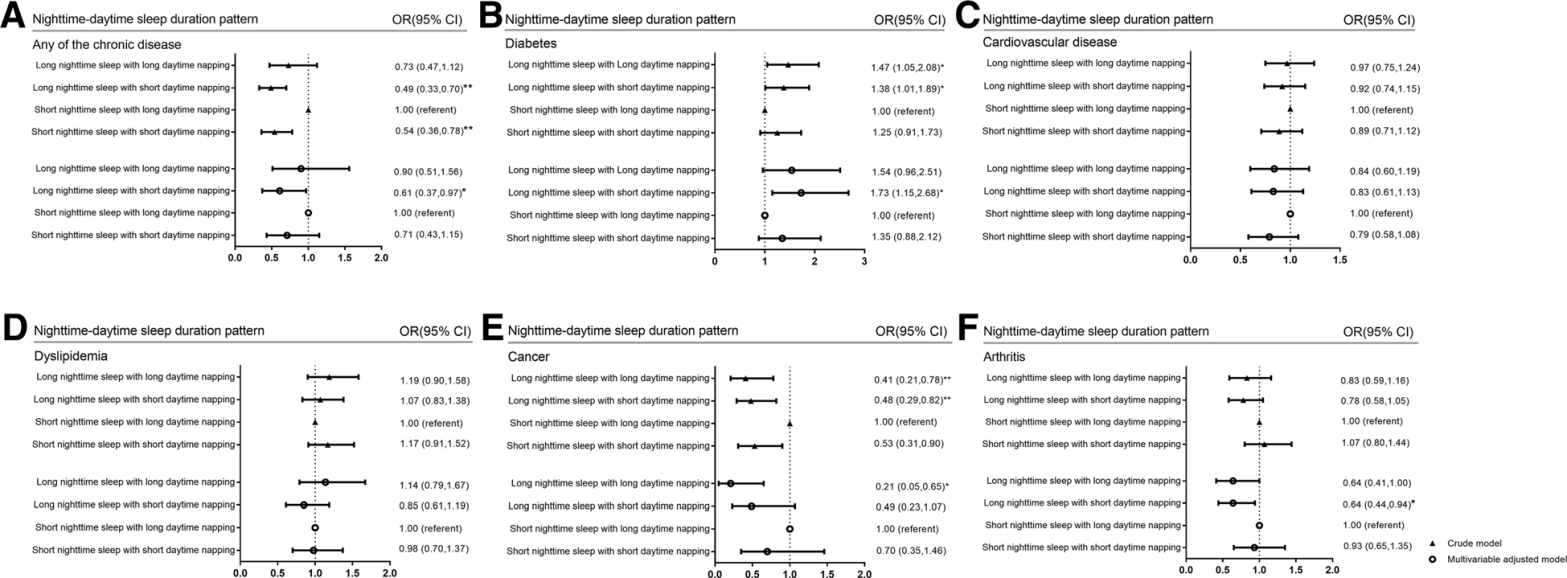
Association between nighttime-daytime sleep duration patterns and prevalence of different chronic diseases in habitual nappers only. Association between nighttime-daytime sleep duration patterns and prevalence of (**a**) any of the chronic disease, (**b**) diabetes, (**c**) cardiovascular diseases, (**d**) dyslipidemia, (**e**) cancer, (**f**) arthritis in the whole population. * *P* < 0.05, ** *P* < 0.01. Multivariable adjusted factors: adjusted for age, gender, BMI, PAL, smoking status, alcohol consumption and arthritis (not adjusted for arthritis in the association between sleep patterns and arthritis

### Association between nighttime-daytime sleep patterns and prevalence of different chronic diseases in habitual nappers only

Among the habitual nappers, we analyzed the nighttime-daytime sleep duration patterns according to nighttime sleep duration and daytime napping duration. The nighttime-daytime sleep duration patterns included: Short nighttime sleep with long daytime napping [*N* = 483 (17.6%)]; Short nighttime sleep with short daytime napping [*N* = 831 (30.3%)]; Long nighttime sleep with long daytime napping [*N* = 511 (18.6%)]; Long nighttime sleep with short daytime napping [*N* = 916 (33.4%)]. In the crude model (Additional file 1: Table S1), the nighttime-daytime sleep duration patterns were significantly associated with prevalence of any of chronic diseases and cancer. Participants with the pattern of “short nighttime sleep with long daytime napping” had the highest prevalence of any of chronic diseases and cancer. For any of chronic diseases, participants with the pattern of “long nighttime sleep with short daytime napping” had the lowest prevalence than others, while the pattern of “long nighttime with long daytime napping” had the lowest prevalence for cancer.

The association between the nighttime-daytime sleep duration patterns and chronic diseases were also analyzed after adjusting for covariates and confounding variables including gender, age, BMI, PAL, smoking status, alcohol consumption [Fig. 3]. In the multivariable adjusted logistic regression model, compared with the reference group of “short nighttime sleep with long daytime napping”, the nighttime-daytime duration sleep pattern “long nighttime sleep with short daytime napping” had lower prevalence of any of chronic diseases [OR and 95% CI, 0.61 (0.37–0.97), *P* = 0.040]. And this pattern has significantly positive association with diabetes prevalence [OR and 95% CI, 1.73 (1.15–2.68), *P* = 0.011] while border-significantly negative association with cancer [OR and 95% CI, 0.49 (0.23–1.07), *P* = 0.072] and significantly negative association with arthritis [OR and 95% CI, 0.64 (0.44–0.94), *P* = 0.021]. However, for CVD, dyslipidemia, no significant associations were observed in the models.

## Discussion

The present cross-sectional study analyzed the prevalence of different chronic diseases by sleep patterns and found some associations between nighttime-daytime sleep patterns and chronic diseases. The prevalence of any of chronic diseases in our study population was 83.8%. CVD had the highest prevalence (64.3%) followed by dyslipidemia (31.3%), diabetes (20.0%), arthritis (14.6%) and cancer (4.3%). We found that compared to those with a sleep pattern of “short nighttime sleep with daytime napping”, those with “long nighttime sleep without daytime napping” had higher prevalence of diabetes and lower prevalence of arthritis. Those with “long nighttime sleep with daytime napping” had higher prevalence of diabetes and lower prevalence of cancer. Then in the habitual nappers, participants were grouped by nighttime-daytime duration patterns, people with “long nighttime sleep with short daytime napping” still had higher prevalence of diabetes, while lower prevalence of any of the chronic disease, cancer and arthritis, and when compared to those with “short nighttime sleep with long daytime napping”.

Inconsistent associations were found between sleep duration and prevalence of diabetes in elderly Chinese. Several cross-sectional and prospective studies suggest a U-shaped association between sleep duration and diabetes [25–28], but one found no association [11]. The relationship between sleep patterns and cancer was also mixed. A meta-analysis of 13 cohorts suggests a positive association between long sleep duration and colorectal cancer, but an inverse association was seen with hormone related cancers, like in the breast [29]. Other studies have shown that short sleep duration, defined as sleeping ≤6 h per night, may be associated with cancers [30–32]. For daytime napping, results were also mixed. A meta-analysis of several prospective studies on the relationship between daytime napping and risk of type 2 diabetes (T2D) found a 17% increased risk of T2D when comparing habitual nappers with non-nappers (RR = 1.17, 95% CI 1.08–1.27) [33]. For excess daytime napping, the meta-analysis evaluated a dose-response relationship, and the results suggested an 11% (95% CI 7–16%) increase in T2D risk for each increment in daytime napping of 30 min/day [33].

With the inconsistent relationship between sleep patterns and chronic diseases, it is important to evaluate the association in a more specific way by considering the inter-relationship between nighttime sleep and daytime napping. However, in our study, there was no significant association between total sleep duration and any of chronic diseases or individual chronic diseases. Significant associations were found between different nighttime-daytime sleep patterns and nighttime-daytime sleep duration patterns. Moreover, we found inverse associations between nighttime-daytime sleep duration patterns and diabetes prevalence as well as cancer prevalence. There was limited research exploring the different effect of sleep patterns on the prevalence of individual chronic diseases. A meta-analysis of 12 studies, involving 130,068 subjects, 49,791 nappers, and 19,059 deaths showed that daytime napping was associated with an increased risk of death from all causes, but no significant association was discovered between daytime napping and cancer mortality [34], indicating some differences in these conditions. Different association of nighttime-daytime sleep patterns with diabetes and cancer can be explained by other factors, such as diet. Diabetes is more susceptible than cancer to the effect of eating habits and nutrient intake [35]. Studies have reported that short sleep duration (often ≤6 h/night) is associated with obesity [36] and diabetes [26]. Insufficient sleep and metabolic disruption may affect a person’s diet and metabolism. Experimental studies and clinical trials showed that sleep restriction had unfavorable impacts on the appetite-related hormones which could further influence energy intake and weight, indicating that chronic metabolic abnormality’s link to chronic disease may be explained by specific sleep patterns [37–40]. For some types of cancer, especially hormone-related cancer, a protective effect of longer nighttime sleep duration was observed. Melatonin had anti-proliferation and anti-angiogenesis effect [41]. Melatonin has been described to be involved in inhibitory influences on sex hormone levels [42], which have been reported to be associated with cancers of the breast, endometrium, ovary, prostate, and thyroid [43]. In addition, melatonin has shown a dose-dependent anti-oxidative effect, acting as a free radical scavenger to provide protection against damage from carcinogenic substance [44]. Melatonin level is positively related to sleep duration [45]. Therefore, longer sleepers may have higher melatonin, which subsequently reduce the risk of certain cancers.

What’s more, we also have some findings on the association between sleep patterns and arthritis. Compare to those with short nighttime sleep and daytime napping/long daytime napping, the pattern “long nighttime sleep without daytime napping/short daytime napping” got both lower proportion of the participants. Results in our study may be consistent with previous researches about the association between arthritis and nighttime sleep duration, which, in details, arthritis was associated with less nighttime sleep [46, 47]. However, little research has focused on the association between arthritis and daytime napping or the sleep pattern combined by nighttime and daytime sleep.

One of the limitations of the present study is that sleep duration was self-reported. Self-reported sleep patterns were susceptible to reporting bias, and objective measures of sleep duration are the best approach. Studying the relationship between subjective and objective measures of sleep, Lauderdale et al. found a moderate correlation between the two measures (*r* = 0.45) and suggested that new questions be added to the commonly used questions [48]. Another limitation of this study is the lack of a temporal relationship. This is an inherent limitation to cross-sectional studies, which only detect associations between variables studied and offer no information on whether the health condition affects sleep or whether sleep is part of the process causing the condition. Besides, few studies have focused on relationship between sleep patterns and pain. However, many studies have found that poor sleep quality was associated with pain, which may be caused by musculoskeletal pain, arthritis, or cancer treatment [49–52]. In our study, it’s really a limitation that we have failed to include questions about detailed pain relating questions. We only had questions about arthritis in our questionnaire and we conceived that we should take consideration of the pain-related problems in our future research. Despite the limitations, our study has several strengths. Firstly, this is a large community-based study, which has limited selection bias. Secondly, our study focused on an elderly population in China. Life expectancy in Shanghai resident was higher than the average level of Chinese people, and in 2015, and that already reached 83.2 [53]. Few studies have focused on a population of with an average age of 74 years old as ours, which includes the older population as well as the older old population. Daytime napping is a common habit among many Chinese in China, and is traditionally viewed as a healthy lifestyle [18]. This practice is different from daytime napping in western countries, where napping is less common and is often unplanned and prompted by sleepiness likely caused by aging, deteriorating health status, or nighttime insomnia [54, 55]. Thirdly, few studies have focused on sleep patterns [56], mostly only on sleep duration or sleep quality. Except the distribution of different sleep patterns, we even found distinct associations between sleep patterns and different chronic diseases, which may be an indication for precision management of chronic diseases. Finally, previous investigations often focused on nighttime sleep duration, which is a commonly surveyed sleep variable in large observational studies. However, we evaluated sleep in four aspects including nighttime sleep, daytime napping, total sleep and the nighttime-daytime sleep pattern.

## Conclusion

We found that nighttime-daytime sleep patterns in our study had distinct relationship with chronic disease, especially for diabetes, cancer and arthritis. Considering that daily sleep habits have important implication in health, further research is needed to improve our understanding on this subject, especially nighttime-daytime sleep patterns.

## Additional file

Additional file 1: **Table S1.** Characteristics of the population by nighttime-daytime sleep duration patterns (among habitual nappers). (DOCX 16 kb)

## Abbreviations

BMI: Body mass index
CVD: Cardiovascular diseases
NAFLD: Non-alcoholic fatty liver diseases
PAL: Physical activity level
