# Supplementary material for: Association between nighttime-daytime sleep patterns and chronic diseases in Chinese elderly population: a community-based cross-sectional study
Source: BMC Geriatr. 2019 Apr 29;19:124. doi: 10.1186/s12877-019-1136-9 (PMC6489270; doi:10.1186/s12877-019-1136-9)
Supplement: Supplementary file 1 — Table S1. Characteristics of the population by nighttime-daytime sleep duration patterns (among habitual nappers). (DOCX 16 kb) [file 12877_2019_1136_MOESM1_ESM.docx]

**Table S1. Characteristics of the population by nighttime-daytime sleep duration patterns (among habitual nappers).**

| **Variables** | **Short nighttime sleep with**  **long daytime napping** | **Short nighttime sleep with**  **short daytime napping** | **Long nighttime sleep with**  **long daytime napping** | **Long nighttime sleep with**  **short daytime napping** | ***P*-value** |
| --- | --- | --- | --- | --- | --- |
| **No. subjects (%)** | 483 | 831 | 511 | 916 |  |
| **Age (years)** | 74.39 (6.87) | 74.66 (6.90) | 74.13 (7.07) | 74.19 (7.28) | 0.481 |
| **Female (%)** | 254 (53.7) | 461 (57.7) | 257 (51.0) | 460 (52.5) | 0.071 |
| **BMI (kg/m^2^)** | 24.19 (3.45) | 24.16 (5.45) | 24.55 (5.54) | 24.34 (3.31) | 0.479 |
| **Physical activity (Met·h/week)** | 31.19 (43.03) | 41.38 (135.86) | 27.77 (26.26) | 36.62 (81.14) | 0.122 |
| **Number of family members** | 2.67 (1.35) | 2.58 (1.29) | 2.48 (1.18) | 2.49 (1.14) | 0.041 |
| **Family income (yuan, RMB)** | 8332.13 (25639.32) | 6750.93 (5035.86) | 7203.68 (10023.30) | 7245.10 (9109.91) | 0.297 |
| **Smoking status (%)** |  |  |  |  | 0.106 |
| Non-smoker | 368 (84.0) | 682 (86.3) | 373 (80.2) | 718 (81.9) |  |
| Ex-smoker | 35 ( 8.0) | 59 ( 7.5) | 51 (11.0) | 88 (10.0) |  |
| Current smoker | 35 ( 8.0) | 49 ( 6.2) | 41 ( 8.8) | 71 ( 8.1) |  |
| **Alcohol consumption (%)** |  |  |  |  | 0.368 |
| None or < once/month | 422 (90.2) | 722 (89.0) | 458 (90.3) | 781 (87.2) |  |
| Past alcohol drinker (>once/month) | 38 ( 8.1) | 75 ( 9.2) | 44 ( 8.7) | 93 (10.4) |  |
| Current alcohol drinker (>once/month) | 8 ( 1.7) | 14 ( 1.7) | 5 ( 1.0) | 22 ( 2.5) |  |
| **Any of chronic diseases (%)** | 359 (90.2) | 606 (83.1) | 376 (87.0) | 671 (81.7) | <0.001 |
| **Diabetes (%)** | 67 (15.1) | 134 (18.2) | 97 (20.8) | 164 (19.7) | 0.126 |
| **CVD (%)** | 283 (58.6) | 464 (55.8) | 295 (57.7) | 518 (56.6) | 0.768 |
| **Dyslipidemia (%)** | 123 (25.5) | 238 (28.6) | 148 (29.0) | 245 (26.7) | 0.502 |
| **Cancer (%)** | 30 ( 7.2) | 28 ( 3.9) | 14 ( 3.1) | 29 ( 3.6) | 0.010 |
| **Arthritis (%)** | 84 (17.4) | 153 (18.4) | 76 (14.9) | 129 (14.1) | 0.066 |

Unless indicated otherwise, data are given as the mean ± SD or as number and percentages.

*Abbreviations:* BMI, body mass index; PAL, physical activity level.
